# Static composting of cow manure and corn stalk covered with a membrane in cold regions

**DOI:** 10.3389/fbioe.2022.969137

**Published:** 2022-09-12

**Authors:** Fengmei Shi, Chengjiao Xu, Jie Liu, Fang Sun, Hongjiu Yu, Su Wang, Pengfei Li, Qiuyue Yu, Dan Li, Xin Zuo, Li Liu, Zhanjiang Pei

**Affiliations:** ^1^ Heilongjiang Academy of Black Soil Conservation and Utilization, Harbin, China; ^2^ Key Laboratory of Combining Farming and Animal Husbandry Ministry of Agriculture, Harbin, China; ^3^ Key Laboratory of Energy Utilization of Main Crop Stalk Resources, Harbin, China; ^4^ College of Resources and Environment, Northeast Agricultural University, Harbin, China; ^5^ Animal Husbandry Institute, Heilongjiang Academy of Agricultural Sciences, Harbin, China

**Keywords:** compost, corn stalk, cow manure, cold region, manure

## Abstract

The disposal of livestock wastes is an urgent task in China. Compost is highly regarded for its ability to treat livestock wastes and protect arable land. In particular, some problems of livestock manure in cold regions, such as low efficiency because of low environmental temperature in winter, urgently need to be solved. In order to provide valuable composting information in the cold area at low environmental temperatures, the composting experiments were carried out with cow manure and corn stalk as substrates. The properties and bacterial community of compost samples in different stages were investigated. The electrical conductivity (EC), total nitrogen (TN), total phosphorus (TP), and organic matter (OM) of the final compost were 551 μS/cm, 1.12, 0.77, and 63.5%, respectively. No *E. coli* or *Ascaris* eggs were detected. The temperature was the key factor to affect the physical-co-chemical and biological properties. The absolutely dominant genera were *Sporosarcina*, *Virgibacillus*, *Flavobacterium*, and *Steroidobacter* in heating, high temperature, cooling, and maturing stages, respectively. Also, these bacteria could act as biological indicators during the composting process. *Cryobacterium*, *Caldicoprobacter*, *Virgibacillus*, and *Sporosarcina* were relatively novel genera in the compost piles in a cold environment. The biodegradation of exogenous substances mainly occurs in the initial and maturing stages. It is proven that composting can be carried out successfully in early spring or later autumn after a harvest.

## 1 Introduction

In recent years, the livestock breeding industry in China has developed rapidly. According to statistics data, the output of cow and poultry in China in 2020 was about 45.65 million and 15.57 billion heads, with an increase of 0.7 and 6.3% year-on-year, respectively (NBSPRC, 2021). A large number of livestock breeding attempts result in a large amount of manure waste. For example, the amount of livestock and poultry manure in China is up to 3.8 billion tons every year, more than the total amount of solid wastes including industrial and agricultural wastes. The comprehensive utilization rate of livestock manure in China was around 76% by the end of 2020 (MARAPRC, 2020). According to the plan of the Ministry of Agriculture and Rural Areas, by 2025, the comprehensive utilization rate of livestock and poultry manure will reach more than 80% (NDRCPRC, 2021). More than 20% of the waste still kept untreated and led to the pollution of water, soil, air, and human health hazards because pathogenic bacteria, parasite eggs, phosphorus, heavy metals, and harmful gases such as hydrogen sulfide, ammonia, and methyl mercaptan could be released from the manure wastes (Míriam et al., 2021; Shen et al., 2021; Duan et al., 2021). The problems of livestock manure pollution have raised extensive concern, and a number of policies have been issued to promote the utilization of stock manure in China (MARAPRC, 2019; MARAPRC, 2021; SCPRC, 2021). Fertilizer use of livestock manure wastes such as composting is the main comprehensive utilization mode (Chang et al., 2019; Onwosi et al., 2017).

Composting is supposed to be a complex biochemical process and is affected by many factors such as composting methods, temperature, C/N, pH, and substrates. Yang et al. (2019) found the nitrogen loss of mixed compost was much higher than that of anaerobic compost, aerobic compost, and farmer’s method. Zhong et al. (2020) studied the bacterial community during the aerobic composting process of dairy manure without any bulking agents and found that *Corynebacterium*, *Bacillus*, *Luteimonas*, and *Nonomuraea* were main functional microbes in different composting phases. *Psychrobacterium* sp., *Pseudomonas* sp., and *Clostridium* sp. were abundant during cow manure composting in the composting facility (Zhao et al., 2013). When rice husk and cow manure were mixed and composted, the unique *Sphaerobacter* and *Myceliophthora* were dominant at high temperatures (Duan and Feng., 2021). It is suggested that the anaerobic compost method might be appropriate for nitrogen retention and less energy input. However, anaerobic composting requires much time because of low efficiency, and the quality of the final product is difficult to be guaranteed (Yang et al., 2019). Methods such as adding microbial agents could facilitate the formation of the humic matter and accelerate the composting process (Abdellah. et al., 2022). Liu et al. (2011) found that the indoor compost piles with microbiological inocula had a more quick temperature elevation, a longer time span of high temperature, and shorter maturation time than natural compost (without microbiological inocula). Duan et al. (2020) studied the effect of *Bacillus subtilis* on carbon components and microbial functional metabolism during cow manure-straw composting, and the results hinted that the addition of *Bacillus subtilis* into the piles could accelerate the compost maturation and improve the final product quality. However, the systematic research on this kind of composting mode at present is few, and aerobic composting has attracted researchers’ attention.

Simple anaerobic compost *in situ* became the main development trend in China. The simple composting methods are commonly used in rural areas. The compost piles are built in the field near the cow farm without ventilation and frequent turnover. Part of the country is in cold regions, and composting time adapted to agriculture planting is at a low-temperature stage. For example, the anaerobic compost is often carried out in the early spring or later autumn in Heilongjiang province. How to promote the composting process and guarantee the end compost quality in cold regions such as Heilongjiang province rich in crop straw and livestock wastes urgently needs to be solved (Shi et al., 2021a; Shi et al., 2021b; Sun, 2019). Hence the feasibility of composting livestock manure in autumn, winter, and early spring in Heilongjiang province was explored. Also, the change and metabolic function of the microbial community during the composting process were studied. So the investigation has more practical significance. It can provide the research basis and data reference for the control of the composting process.

## 2 Material and methods

### 2.1 Composting material and methods

The cow manure was taken from a cow farm in Heilongjiang province. The corn straw was purchased from the farmers near the research base and crushed to 0.5–3.0 cm. The basic physical and chemical properties of the raw materials are shown in Table 1. The substrate was dried to a constant weight in a hot air circulation oven at 105°C, and the water content (WC) was calculated according to the weight before and after drying. Total nitrogen (TN) and organic matter (OM) were evaluated according to the methods specified in NY/T 1121.24-2012 and NY/T1121.6-2006. The OM divided by 1.724 is the total organic carbon (TOC) value.

**TABLE 1:** Basic physicochemical properties of cow manure and corn stalks.

Substrate	WC (%)	TOC (%)	TN (%)	C/N
Cow manure	84.33	39.00	2.06	18.93
Corn stalk	15.87	41.18	0.92	44.76

The C/N and the water content of the composting mixture were about 30:1 and 60%, according to the research studies and our laboratory compost results (Sánchez et al., 2017; Macias-Corral et al., 2019). Here, 1.592 tons of cow dung, 2.556 tons of grounded straw, and 1.852 tons of water were mixed. Then, the mixture was divided into three equal portions, which were piled up into three semi-cylindrical compost strips with a forklift on the waterproof concrete floor and covered with a 2.0-mm thick HDPE (high-density polyethylene) membrane. Three thermocouples were inserted into the front, middle, and rear of the compost pile, respectively. The experiments were carried out in an organic waste treatment plant in Heilongjiang province from March to May, lasting 70 days. The piles were turned over on the 30th day using a front loader.

### 2.2 Sampling and index measurement

The temperature of compost piles and the environment were measured and recorded daily, which could be viewed on a mobile phone after the construction of the piles. According to the composting pile temperature, N1, N2, N3, and N4 were sampled on the 10th day (heating stage), 20th day (high-temperature stage), 50th day (cooling stage), and 70th day (maturing stage). The method of multi-level and multi-point sampling was adopted, sampled compost was then fully mixed, and 500 g of fresh compost was collected in the same pile. Then, the collected samples from the three different piles were mixed again. Some fresh samples were stored at 4°C to measure the WC, pH, and electrical conductivity (EC). The pH and EC levels were measured in the extraction liquid of fresh compost (1:10 of compost to deionized water, v/m) using a pH meter and a conductivity meter, respectively. The methods were described by previous studies (Shi et al., 2021a; Ding et al., 2020). At the same time, the color and smell of each group were investigated. About 50 g of fresh samples were stored at −80°C ready for 16S rRNA analysis. The remaining parts were dried at room temperature and used to test TN, TP, and OM, according to the methods specified in NY/T 1121.24-2012, NY/T 88-1988, and NY/T1121.6-2006, respectively.

### 2.3 16S rRNA analysis

DNA (deoxyribonucleic acid) was extracted using the FastPrep DNA kit (QBIOGENE, United States), according to the kit instructions, and the extracted DNA was detected by 1% agarose gel electrophoresis. Then, 30 ng DNA samples were extracted from the qualified compost samples as PCR (polymerase chain reaction) templates. The universal primers (338F/806R) were used to PCR-amplify the V3-V4 area of 16S rDNA. The general primer sequences of 16S rRNA were as follows: F: 338F (5′-ACT​CCT​ACG​GGA​GGC​AGC​AG-3′) and R: 806R (5′-GGACTACHVGGGTWTCTAAT-3′). PCR reaction conditions were as follows: 95°C for 3 min; 95°C for 30 s, 55°C for 30 s, 72°C for 45 s, 27 cycles; 72°C for 10 min. PCR amplification products were purified using Agencourt AMPure XP magnetic beads, dissolved in elution buffer, and labeled to complete the database building. The fragment range and concentration in the database were checked using the Agilent 2100 Bioanalyzer. The qualified database was sequenced and analyzed on the HiSeq platform. Then, the microbial function was predicted with the PICRUSt program, described in our previous study (Shi et al., 2021b).

### 2.4 Data analysis

Origin software was used to process data, plot, and calculate Pearson correlation coefficients and probability.

The Pearson correlation coefficient (r_p_) describes the degree of linear correlation between two variables. A larger absolute value of r_p_ indicates a better correlation. The r_p_ between variable x and variable y can be calculated as follows:

$$r_{p}=\frac{\sum \left(x-\bar{x}\right)\left(y-\bar{y}\right)}{\sqrt{\sum {\left(x-\bar{x}\right)}^{2}\sum {\left(y-\bar{y}\right)}^{2}}},$$

where x and y are the variables, and $\bar{x}$ and ȳ are the average of the variables x and y, respectively. Then, the function t is used to evaluate the significant p.

$$t=\frac{r_{p}}{\sqrt{\left(1-r_{p}\right)}/\sqrt{n-2}},$$

where n is the number of variables x or y. The p-value can be obtained easily *via* the t-distribution table.

## 3 Results

### 3.1 Changes of parameters of compost

#### 3.1.1 Temperature

**FIGURE 1:**
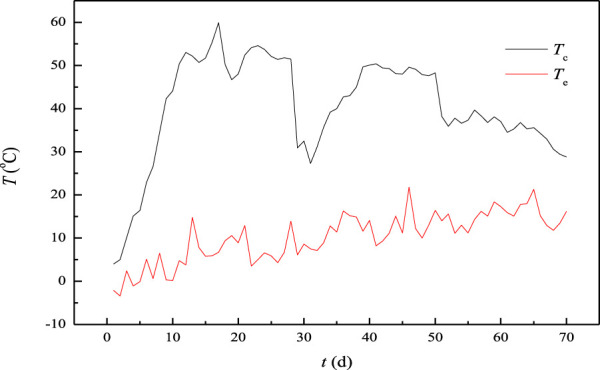
Temperature of the compost pile and environment.

The temperature profiles of the compost piles were much different with different compost modes or manual management. For example, frequent turning would lead to frequent temperature rise and fall of the compost piles, and more peaks would be found on the temperature curves [shen et al., 2019]. However, all the temperature profiles consisted of heating, high temperature, and cooling parts [Duan et al., 2020; Duan et al., 2021] The temperature during the composting process is shown in Figure 1. The temperature profiles were also multi-peaked, and composting consisted of heating, high temperature, and cooling stages. It can be seen that the temperature rose rapidly at the rate of 4.74 °C/d and reached 44.1°C in 10 days, 50.4°C in 11 days, and 59°C in 17 days, the first temperature peak. After 20 days, the temperature decreased to 48°C because of the fall in environmental temperature and then quickly recovered to above 50°C. The temperature of the composting pile decreased to about 30°C due to the heat loss because of the pile turning on the 30th day. After turning, the oxygen concentration in the compost pile increased, and the microorganisms became active gradually after adapting to the new environment. Then, the temperature of the compost pile increased at a rate of 1.9°C/d and reached 50°C on the 39th day, forming the second peak. The temperature decreased slightly at the rate of −0.19°C/dfrom the 39th to 50th day. The temperature drop was very small and negligible. The temperature dropped rapidly from 48.3°C on the 50th day to 38.2°C on the 51th day. The temperature was closely related to the metabolism of organic compounds by microorganisms and the heat loss to the environment (Schueler et al., 2021; Khalil et al., 2001). In the early stage of composting, there were abundant easily degradable organic materials such as starch, and protein, used by microorganisms for reproduction and metabolism activities. The number of microorganisms increased rapidly. A great deal of biological heat was produced, and the temperature of the pile quickly rose when the accumulated biological heat was far more than the heat lost to the environment. When the number and taxonomy of microorganisms became stable, the temperature also became stable, and compost was in the thermophilic stage. The temperature gap between the local ambient and the compost pile temperatures was much greater in the two thermophilic stages, with most of the degradable materials being degraded and utilized during the first and second high-temperature stages. The available materials in the compost pile were not enough to meet the needs of the microbial community. The growth and reproduction of microorganisms were then inhibited, the produced bio-heat decreased correspondingly, and the temperature of the compost pile decreased continuously at the rate of 0.52°C/d with the heat radiation from the reactor to the ambient.

There are a lot of pathogenic bacteria and parasite eggs in the feces of livestock and poultry, which affect the safety of land use. The pathogenic bacteria and parasite eggs were proved to be temperature-sensitive (Wichuk and McCartney, 2007; Heck et al., 2013). Most *E. coli*, *Salmonella* and *Shigella* bacteria will be killed for an hour at 55 C. If the temperature is kept for 15–20 min at 60°C, all of them will be eliminated. In composting environments, it will take a long time to obtain the same results. It took about 4 days to inactivate *Salmonella* when the composting temperature was higher than 55°C [Marilyn et al., 2009; Macias-corral et al., 2019]. *E. coli* could be eliminated in 25 days when the composting temperature was between 45 and 55°C (Wichuk and McCartney, 2007; Macias-corral et al., 2019). As a result, certain requirements for composting temperature and duration were proposed in order to meet the hygienic standards. According to the requirements of the “Technical Code for Composting of Livestock and Poultry Manure” (NY/T3442-2019) and “Technical Code for Harmless Treatment of Livestock and Poultry Manure” (GB/T36195‐2018), the time span should be kept more than 14 days above 45°C for strip composting. It was 18 days from 50.4°C on the 11th day to 51.5°C on the 28th day in this experiment, which ensured the health and safety of the land use of the final compost product.

#### 3.1.2 Indexes of composting

With the undergoing of composting, the WC of the compost decreased from 60.30% of N1 to 43.40% of N2 and then increased to 45.49% of N3 because of addition of water. When the composting was completed, the WC of the end product became 42.1%. The changes in pH were closely related to the metabolic activity of microorganisms in the compost piles. At the initial stage of composting, the composting material was basically neutral, and the pH value was 7.27. Then, the pH value increased to 9.29 due to the emission of NH_3_ from protein and the accumulation of NH_4_^+^ in the composting matrix (Zhong et al., 2020). Then, microbial organisms used carbohydrates from biodegradable organic substances such as hemicellulose and cellulose to produce organic acids by metabolic activities. When the content of these acids was much higher than that of NH_3_, the pH value kept decreasing and was 8.23 at the end of composting (Duan et al., 2021). The changes in pH values during the composting process were consistent with previous research studies (Liu et al., 2011; zhong et al., 2020). The EC of N1 was at a maximum of 5.390 ms/cm because the mineral salts were released or ammonium salts were formed (Duan et al., 2021). At the end of composting, the EC of the compost became 551 μS/cm. It was much lower than the suggested value of 4 dS/m and meant the final compost was safe for use (Sánchez et al., 2017). TN, TP, and OM of the final compost were 1.12%, 0.77%, and 63.5%, respectively. *E. coli* and *Ascaris* eggs were not detected in the final product. The compost could be used as an acid soil improvement and conditioning agent, as well as the cultivation of camptothecin crops. It should not be used in saline-alkali soil, saline soil, and crops and vegetables that were sensitive to low pH. The indexes are summarized in Table 2.

**TABLE 2:** Some properties of the compost.

Index	N0	N1	N2	N3	N4
WC(%)	60.30	48.65	43.40	45.49	42.1
pH	7.27	9.29	8.95	8.72	8.23
EC (uS/cm)	1,690	5,390	3,560	356	551
TN (%)	1.45	0.64	1.3	1.26	1.12
TP (%)	1.69	0.54	0.88	0.92	0.77
OM (%)	68.5	67.3	65.1	64.6	63.5

### 3.2 Succession of the bacterial community during composting

The composting process is carried out with the cooperation of many kinds of microorganisms. The composition and succession of microorganisms are affected by T, pH, and WC. In order to study the change in the microbial community of static composting in a cold region and provide support for screening high-efficient composting bacteria or developing special composting bacteria agents, the bacterial community was studied *via* 16S rRNA.

#### 3.2.1 Venn diagram

**FIGURE 2:**
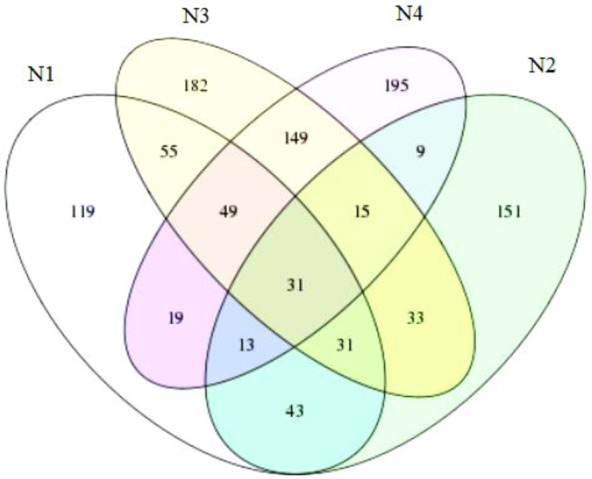
Venn diagram.

From the Venn diagram (Figure 2), it could be seen that the number of OTUs was N3 > N4 > N1 > N2. The common OTUs of N1, N2, N3, and N4 were 31, accounting for 8.61%, 9.51%, 5.69%, and 6.46% of their own total OTUs, respectively. Most of the OTUs in the samples were unique. Zhong et al. (2020) and Wang et al. (2018) gave similar reports. The overlapping OTUs of N2 and N4 were the least, accounting for 20.86% and 14.16% of the total OTU numbers of N2 and N4. The overlapping OTUs of N1 and N3 were the most, accounting for 31.11% and 23.33% of the total OTU numbers of N1 and N3. N1 and N2 shared 32.8% and 36.2% of the total OTU numbers of N1 and N2, respectively. N2 and N3 shared 33.7% and 20.2% of the total numbers of N2 and N3 OTUs, respectively. N3 and N4 shared 110 OTUs, which accounted for 44.8% and 50.8% of the total OTU numbers of N3 and N4, respectively. It indicated that the composting system had its own survival mechanism, and only a small number of bacteria in the whole composting process could be detected because of their good adaptability to the composting environment and great resistance to unfavorable conditions. Although these bacteria cannot be used as indicator organisms of the heating, high temperature, cooling, and maturing stages of composting, high-performance bacteria could be screened out to improve the composting efficiency or accelerate the composting process by adding them at the beginning of composting in tough composting conditions. On the other hand, the degradation of different organic compounds corresponds to different microbial communities. Therefore, the succession of microbial communities happened in different composting stages.

#### 3.2.2 Rarefaction curve

**FIGURE 3:**
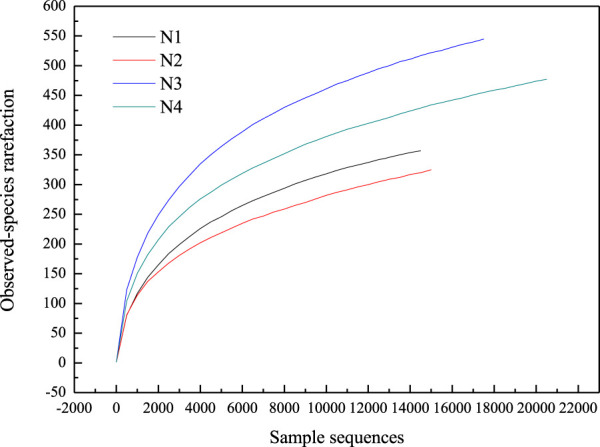
Observed-species rarefaction.

The rarefaction curve can reflect the authenticity of the test results (Ding et al., 2020; Li and Xu., 2015). The observed-species index increased rapidly before 4,000 sample sequences and then increased slowly (Figure 3). The coverage of the four samples was higher than 0.99. The observed-species and coverage indexes indicated that the bacteria in each sample had been detected, the detection results were true. The indexes of Chao and Ace can indicate the community richness and evenness of samples, while the information on community diversity can be obtained from Shannon and Simpson rarefaction indexes (Zhong et al., 2020; Lei et al., 2021). The indexes of different rarefaction curves are summarized in Table 3. It could be seen that the indexes of Chao and Ace decreased in the order of N3, N4, N2 and N1. N1 and N3 had the lowest and highest bacterial abundance, respectively. The value of Shannon and Simpson indexes hinted that N3 had the maximum bacterial community diversity, then followed by N4, N1 and N2. The Chao index of N1 and the Shannon index of N2 were the smallest. The composting started below 10°C, and the bacteria in compost substrates were psychrophilic. Most of them were inactivated when the temperature increased rapidly and led to the least biological abundance of N1. When the temperature up to 50°C was kept for nearly 10 days, some microorganisms adapted to the compost environment and produced, leading to more biological richness in sample N2 than N1. However, there were fewer thermophilic bacteria above 50°C, and the diversity of bacteria was the least in N2. The alpha diversity of bacteria had some differences compared to those reported by Zhong et al. (2020). They reported much larger Chao and Shannon indexes. The Chao index of the samples in the thermophilic stage and the Shannon index of the sample in the cooling stage were the smallest. It can be explained by the temperature changes during the composting process. The reported composting by Zhong et al. (2020) was carried out at 28.03°C, much higher than ours. The bacteria in compost substrates were mesophilic, and the effect of high temperature on such bacteria was less than that of psychrotrophic bacteria. Hence, the initial temperature of composting would affect the bacterial performance.

**TABLE 3:** Indexes of different rarefaction curves.

Sample	Chao	Shannon	Simpson	Ace	Coverage
N1	479.936	3.407	0.0758	461.854	0.9926
N2	496.500	3.405	0.0861	574.348	0.9923
N3	696.000	3.978	0.0746	689.282	0.9914
N4	647.787	3.812	0.0596	639.175	0.9930

#### 3.2.3 Diversity of the microbial community

**FIGURE 4:**
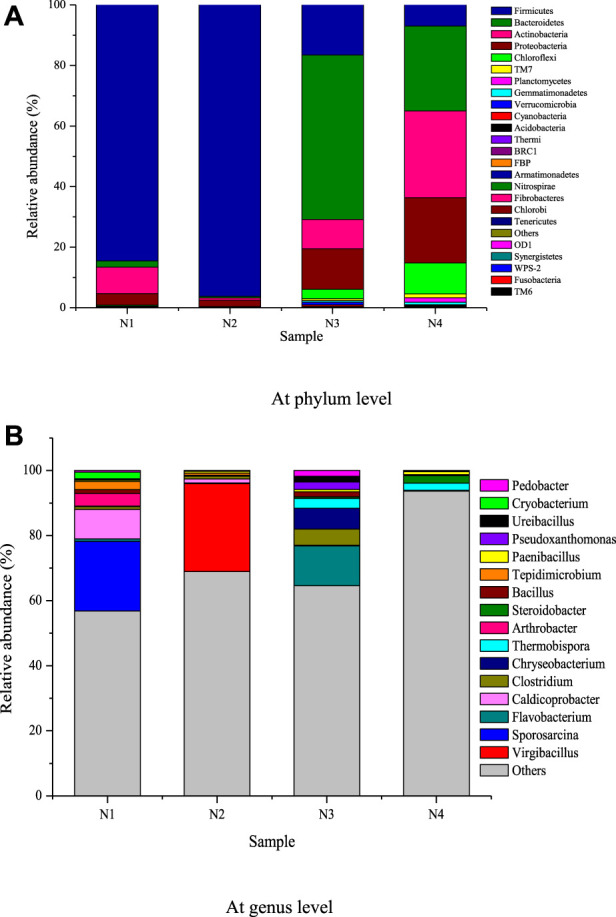
Barplot of bacterial relative abundance. **(A)** At phylum level. **(B)** At genus level.

A total of 33 phyla and 16 genera were detected in the four samples, shown in Figures 4A,B. The dominant phyla (relative abundance >1%) were four in N1, two in N2, five in N3, and 7 in N4. It can be seen from Figure 4A that Proteobacteria, Actinobacteria, Bacteroidetes, and Firmicutes were the dominant phyla in N1, with relative abundance of 3.82%, 8.79%, 1.96% and 84.6%, respectively. Proteobacteria (2.08%) and Firmicutes (96.2%) still remained dominant in N2. In N3, the dominant phyla included Chloroflexi (3.16%), Proteobacteria (13.36%), Actinobacteria (9.63%), Bacteroidetes (54.32%) and Firmicutes (16.56%). In N4, the relative abundance of Planctomycetes, TM7, and Chloroflexi increased dramatically to 1.51%, 1.23% and 10.22%, respectively. The relative abundance of Proteobacteria, Actinobacteria, and Bacteroidetes was about 21.52%, 28.65% and 28.06%, respectively. The number of dominant phyla decreased with the elevating temperature (N1 to N2), then recovered after a turnover (N3) and increased in the maturing stage (N4) at a lower temperature than the other composting processes. It hinted that composting could be completed successfully with the cooperation of different microorganisms. The microbial community structure and abundance were much different in the initial, thermophilic, cooling, and maturing stages of composting. But only a few microorganisms were desired. For example, Proteobacteria, Actinobacteria, Bacteroidetes, and Firmicutes were often detected to be important in composting environments (Shen et al., 2019; Sánchez et al., 2017; Zhong et al., 2020; Duan et al., 2021). In our study, the absolutely dominant phyla were Firmicutes in N1 and N2, Bacteroidetes in N3, and Actinobacteria in N4, which played a key role in the degradation of the complex organic matrix. However, Chloroflexi and Planctomycetes emerged in N3 and N4 could be explained by their slow growth and act as indicator bacteria (Zhong et al., 2020).

The bacteria at the genus level in the four samples are shown in Figure 4B. The relative abundance of *Cryobacterium*, *Tepidimicrobium*, *Bacillus*, *Arthrobacter*, and *Sporosarcina* in N1 was 2.05%, 2.53%, 1.17%, 3.81%, and 21.33%, respectively. *Caldicoprobacter* (1.24%) and *Virgibacillus* (27.05%) were dominant in N2. *Pedobacter*, *Ureibacillus*, *Thermobispora*, *Bacillus*, *Chryseobacterium* and *Flavobacterium* in N3 became dominant genera with the relative abundance of 1.81%, 1.57%, 3.05%, 1.56%, 6.35%, and 12.2%, respectively. In N4, *Thermobispora* (2.21%) was still kept dominant, and Steroidobacter (2.29%) appeared to be dominant for the first time. The absolutely dominant genera were *Sporosarcina* in N1, *Virgibacillus* in N2, *Flavobacterium* in N3, and *Steroidobacter* in N4. Compared with the existing studies, the dominant bacteria in our compost samples such as *Cryobacterium*, *Caldicoprobacter*, *Virgibacillus*, and *Sporosarcina* were relatively novel (Lei et al., 2021; Duan et al., 2020; Zhong et al., 2020). The differences could be explained by the initial temperature of the compost substrates and the compost piles. In sample N1, psychrophilic bacteria of *Cryobacterium*, *Sporosarcina*, and *Arthrobacter* and mesophilic bacteria of *Tepidimicrobium* coexisted because of the lower temperature of the initial composting substrates. *Caldicoprobacter* and *Virgibacillus* could endure higher temperatures and had high relative abundances. *Flavobacterium* was often reported during composting of organic wastes containing high lignocellulose (Li et al., 2019). *Steroidobacter* could be an indicator of the completion of composting.

### 3.3 Relationship between bacterial and compost indexes

According to the Pearson correlation coefficients with probability, about 30% of the phyla were sensitive to the temperature of compost piles. Chloroflexi, Planctomycetes, FBP, Chlorobi, OD1, Synergistetes, and TM6 were significantly negatively correlated with the temperature at the 95% confidence level. The correlation coefficients (p) of Chloroflexi, Planctomycetes, and FBP were −0.95999 (0.04001), −0.98486 (0.01514), and −0.9629 (0.03703), except that Chlorobi, OD1, Synergistetes, and TM6 were all −0.98334 (0.01666). The relative abundant phyla of Proteobacteria, Actinobacteria, Bacteroidetes, and Firmicutes in the compost piles could tolerate a certain high temperature, and their correlation coefficients (p) were −0.85301 (0.14699), −0.89596 (0.10404), −0.25194 (0.74806), and 0.67556 (0.32444), respectively. Nitrospirae was negatively associated with TN during composting, which was significant at the 95% confidence level with correlation coefficients and probability of −0.9283 and 0.03291, respectively. Therefore, the succession of bacteria was mainly affected by temperature. The species and abundance of bacteria were related to their tolerance to temperature. At the genus level, Paenibacillus had a significant negative correlation with pH and OM content (p < 0.05). *Arthrobacter* and *Sporosarcina* had significant negative correlations with TN content in the compost (p < 0.05), and *Steroidobacter* had a significant negative correlation with T at the 95% level (p = 0.026). The results, in turn, explained the succession of the microorganism during the composting process.

### 3.4 Functional prediction

**FIGURE 5:**
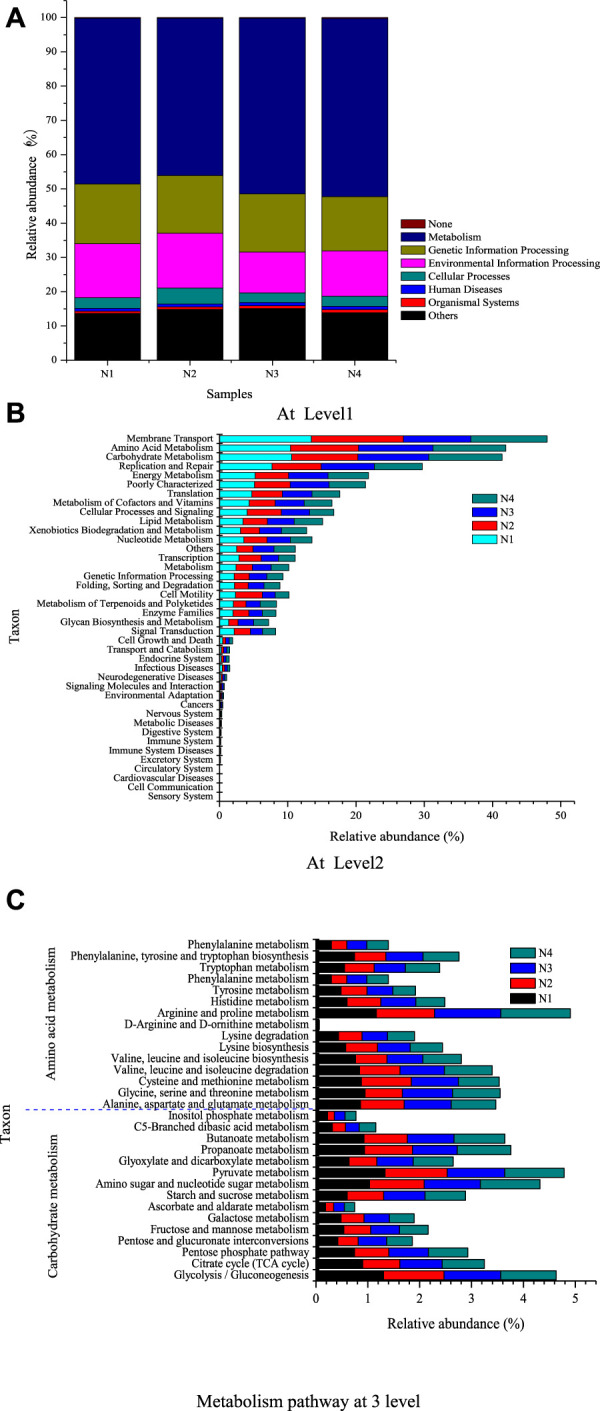
Functional prediction. **(A)** At level 1. **(B)** At level 2. **(C)** Metabolism pathway at level 3.

The metagenomic function was predicted by the PICRUSt program at three levels, and the results are shown in Figures 5A–C. It can be seen from Figure 5A that 48.42%, 45.95%, 51.25%, and 52.03% of genes related to microbial metabolism were found in N1, N2, N3, and N4, respectively, which were the most relative abundance among the eight metabolic pathways. The microbial functional pathways at level 1 including cellular processes, environmental information processing, and genetic information processing metabolism had been further classified into 40 metabolic pathways shown in Figure 5B. Membrane transport was the most abundant metabolic type in N1, N2, and N4 with a relative abundance of more than 10%. If membrane transport was not considered, the first abundant metabolic pathway was amino acid metabolism, in which arginine and proline metabolism relating to the nitrogen metabolism, CO_2_, and organic acid products had an absolute advantage (Kiupakis and Scneider., 1998; Wang et al., 2018). The relative abundance of carbohydrate metabolism closely followed that of amino acid metabolism. The relative abundance of genes involved in pyruvate metabolism and glycolysis/gluconeogenesis was almost similar. Pyruvate metabolism played a pivotal role in the metabolic connection of carbohydrates, amino acids, and lipids. The pyruvate metabolism and glycolysis/gluconeogenesis pathway were responsible for the organic acids such as propanoate and butanoate, which led to the variable pH value (Duan et al., 2020). The relative abundances of genes related to xenobiotic biodegradation and metabolism were 3.06% in N1, 2.80% in N2, 3.23% in N3, and 3.67% in N4. It hinted that biodegradation of exogenous substances mainly occurred in the initial and maturing stages. In the early stage of composting, organic matter was degraded, while humus and other macromolecules were formed in the maturing stage.

According to the Pearson correlation coefficients with probability, *Thermobispora* was significantly positively correlated with glycan biosynthesis and metabolism and metabolism at the 95% confidence level with the correlation coefficients (p) of 0.98572 (0.01428) and 0.97035 (0.02965), respectively. There was a significant positive correlation between the bacterial species of *Steroidobacter* and the metabolism of terpenoids and polyketides with a p-value of 0.045, only a little less than 0.05. Except for these two genera, p-values of *Paenibacillus* between lipid metabolism and nucleotide metabolism were 0.06805 and 0.05205 with the correlation coefficients of 0.93195 and −0.94795, respectively. *p*-values of *Steroidobacter* between xenobiotic biodegradation and metabolism was 0.06396. The Pearson correlation coefficient of *Virgibacillus* and carbohydrate metabolism was −0.94973 with a *p*-value of 0.05027.

## 4 Discussion

*Steroidobacter* is a genus of the Clostridium phylum. It can degrade cellulose with endoglucanase, exoglucanase, and xylanase (Zhang et al., 2014). However, *Thermobispora bispora* can produced glucaric acid from the hemicellulose substrate by secreting uronate dehydrogenase which can exhibit more than 58% of the activity after 1 h at the temperature of 60°C and pH 7.0–7.5 (Li et al., 2018). It had good cellulose degradation activity by binding secreting endoglucanase to Ser131, Met263, Gln298, and His310 of cellulose (Paul et al., 2020). Some researchers thought it could play a dominant role in chemical metabolism and mutual nutrition (Perez et al., 2021). *Sporosarcina* can produce urease, closely related to the nitrogen cycle (Alves et al., 2022; Mahdi et al., 2021), while *Virgibacillus* can secrete amylase and protease, which are related to the degradation of protein and starch (Satabdi et al., 2020). It is also related to sulfur metabolism. *Flavobacterium* is a strictly aerobic bacterium and has high degradation activity to cellulose (Vikas et al., 2018: Di Maiuta et al., 2013). It can produce acids by fermenting glucose, fructose, and maltose.

At the initial stage of composting, easily hydrolyzable organic matter, such as protein, urea, and starch was first biodegraded and utilized. Hence Sporosarcina was the dominant strain associated with the utilization of the metabolites from protein. *Cryobacterium* associated with starch hydrolysis and *Tepidimicrobium* associated with the metabolism of hemicellulose, *Caldicoprobacter* utilizing sugars generated from starch and hemicellulose metabolism, and *Arthrobacter* related to the sulfur element from protein metabolism were more abundant Therefore, protein hudrolysis and metabolism mainly occured in this stage. In the high-temperature stage (N2), most of the genera of Actinobacteria, Bacteroidetes, and Proteobacteria were inhibited. But *Virgibacillus* in Firmicutes could adapt to the high temperatures in this stage and then hydrolyze organic compounds such as starch and hemicellulose, as well as utilize the metabolites of the bacterial community. In the N3 stage, the temperature of the compost pile dropped to about 40°C, and most bacteria were thermophilic. Chloroflexi (3.16%), Proteobacteria (13.36%), Actinobacteria (9.63%), and Bacteroidetes (54.32%) returned to the dominant phyla, except that the relative abundance of Firmicutes was still high. *Flavobacterium* in Bacteroidetes became the overwhelming dominant genera, so cellulose degradation and metabolite utilization occurred in this stage. In the maturing stage, *Steroidobacter* of Proteobacteria, a denitrifying bacterium, became a new dominant genus. It hinted that metabolism associated with denitrification mainly happened in this stage. It was believed that lignin degradation could be ignored below 28°C or above 75°C (Tuomela et al., 2000). So the degradation of lignin may be in high temperature and cooling stages.

Research studies showed that adding bacteria or bacterial flora screened from the composting environment could prolong the high-temperature stage of composting and improve the composting efficiency (Sánchez et al., 2017; Li et al., 2019; Duan et al., 2020). In our study, *Virgibacillus* and *Caldicoprobacter* could be inoculated to improve the composting efficiency at low environmental temperatures. It was practical to identify the composting stages by biological indicators such as *Sporosarcina*, *Virgibacillus*, *Flavobacterium*, and *Steroidobacter* in the initial, thermophilic, cooling, and maturing stages of composting with cow manure and corn stalk at cold ambient temperature, respectively.

## 5 Conclusion

The properties and bacterial community of the static composting in the cold area at low environmental temperatures with cow manure and corn stalk as substrates were investigated. It was proved that the composting could be carried out successfully in early spring or later autumn after the harvest. The end products can meet the requirements of the relevant national safety and health standards. The succession of microbial communities could be observed in the composting process. The absolutely dominant phylum was Firmicutes in N1 and N2, Bacteroidetes in N3, and Actinobacteria in N4, respectively. And they were playing key roles in the degradation of the complex organic matrix. The absolutely dominant genus was *Sporosarcina* in N1, *Virgibacillus* in N2, *Flavobacterium* in N3, and *Steroidobacter* in N4. The bacterial flora including *Sporosarcina*, *Virgibacillus*, *Flavobacterium*, and *Steroidobacter* could be used to improve the composting efficiency. The biodegradation of exogenous substances mainly occurred in the initial and maturing stages. The functional genes of amino acid metabolism and carbohydrate metabolism were abundant and could reflect the changes of N and organic acids during the composting process.

## Data Availability

The raw data supporting the conclusion of this article will be made available by the authors, without undue reservation.
